# A Carr off the Track

**Published:** 1884-07

**Authors:** Geo. E. Blackham


### A CARR OFF THE TRACK.

GEO. E. BLACKHAM, M. D.

With all due deference to my good friend, Doctor Carr, with all respect for the right of every man to have his own opinion and to express it. I "feel moved" to enter my emphatic protest against the doctor's article on homœopathy and allopathy in the April number of THE BISTOURY, which starting from false premises, leads by false logic to the false conclusion of a therapeutic nihilism. The argument seems to be "We don't know anything about anything, therefore let us do nothing lest we do harm." I respectfully decline to follow my good friend into any such medical slough of despond, out of which, however, he is careful to keep in practice as many of his grateful patients can testify to this day.

Medicine is not an exact science, the elixir vitæ is still unattainable, the fountain of youth has eluded the search of Prince de Leon and his followers to this day. It is true, but much real advance has been made, human life is longer, more endurable, happier than ever before, for much of this amelioration of existence, medicine and medical men are directly responsible to a degree much greater than the world at large gives them credit for. It is too much the fashion in these unbelieving days to run down "the doctors," because they can not work miracles, and to denounce them as empirics, charlatans and imposters. It is bad enough to have to endure this maltreatment at the hands of the ignorant and prejudiced among the laity, but when a valued and valuable member of the profession furnishes stones for the aforesaid section of the laity to throw, it is time to enter a protest and to show the errors into which he has fallen.

The first error is the assumption that the medical world, (meaning by this the practitioners of medicine) are divided into two great and totally opposed schools, the homœopathic and the allopathic. That there is a large school of physicians who claim to *base* their practice upon the dogma of *similia similibus curantur*, and may therefore be fairly labeled homœopathic is not to be denied. But where is the school which claims to base its practice upon the opposite dogma, and may therefore fairly be labeled allopathic? So far from there being such a school, I doubt that there is even a single physician who claims to base his practice upon this latter dogma, at least I never met one, and my acquaintance with physicians has been reasonably extensive.

The next error in Dr. Carr's premises is the assumption that if there are two such schools, one or both *must* be *all* wrong. It is quite possible, to say the least, that both schools may be part wrong and part right. No particular school or class holds a patent right upon truth and wisdom, so that none others can share these precious things, nor is any school so infallible that some error may not creep into its creed or practice.

When one comes to look into the question of practice, the difference between the practitioners of the regular or old school, (which I assume is what Dr. Carr means by the allopaths,) and those of the homœopathic school is not so wide as Dr. Carr seems to assume. In the general care of the patient, the direction of hygenic conditions of food, clothing, ventilation, etc., etc., there is in the main an agreement among the more intelligent practitioners of all schools, and even in the selection of drugs the correspondence is quite as remarkable as the difference, though in the explanation of the reason *why* the particular drug was chosen may differ widely.

When Doctor Carr abandons for the time his medical agnosticism and becoming a dogmatist, lays down the law thus: "All organic diseases are totally incurable by any or all systems of medication, and the reason is that they have no inherent tendency towards spontaneous recovery; *only such diseases are curable as tend to cure themselves;*" does he not get way off the track of observed fact ? And would not the fair inference of his dogma be, if it were true, that he and all the rest of us who practice or (pretend to practice) medicine are vain, ignorant, mischievous pretenders impotent for good and powerful for harm.

But happily the doctor's dogma is not true. Let us take a very common and comparatively simple disease, selected because the diseased parts are easily inspected—viz: Trachoma, or, "Granulated Eyelids." Is this a purely functional disorder? Do not the new growths upon the lining membrane of the lid, the new blood-vessels that spread over the irritated cornea and obscure vision, constitute an organic lesion—and has it a tendency toward spontaneous recovery? And yet how often do we see recovery take place under the proper application of drugs, amendment taking place from day to day and keeping pace with the treatment, halting when it is stopped and advancing again when it is resumed and as clearly due to the drug action as any effect is to any cause that the human mind can take cognizance of.

Who that has seen the lamentable effects of untreated iritis, the agony, the contracted pupil firmly glued to the lens by inflammatory adhesions, and the resulting blindness, total or partial, and, on the other hand, has witnessed the readiness with which most cases of this disease yield to the influence of Atropia, can fail to believe that here is another disease which does *not* tend to cure itself and yet is curable by drugs properly selected and administered.

It is not necessary to multiply instances though it might easily be done in support of the statement that diseases which do not possess an inherent tendency to recovery can be cured. It may be true that in many cases we cannot explain *why* a drug has certain effects but so long as we know *how* it will act it is a matter of sentiment rather than practical utility to search out the *why*.

The blind see, the deaf hear, the lame walk, anesthesia flings the blessed boon of unconsciousness over the sufferings of the operating room and the maternity chamber, life is lengthened and suffering lessened on every side by efforts of medical science made wise by *experience*. Far be the day when for empiricism, the experimental method, which has given such results in this as in all other fields of science, shall be substituted the dicta of the dogmatist or the *a priori* reasoning of the metaphysician.

